# Multifocal epithelial hyperplasia: an understudied infectious disease affecting ethnic groups. A mini review

**DOI:** 10.3389/fcimb.2024.1420298

**Published:** 2024-07-25

**Authors:** Laura Conde-Ferráez, María del Refugio González-Losa

**Affiliations:** Laboratorio de Virología, Centro de Investigaciones Regionales, Universidad Autónoma de Yucatán, Mérida, Mexico

**Keywords:** human papillomavirus, oral disease, HPV13, indigenous population, viral infection, pathology

## Abstract

Focal Epithelial Hyperplasia or Multifocal Epithelial Hyperplasia (MEH), also known as Heck’s disease, is considered a rare pathology of the oral mucosa associated with human papillomavirus types 13 and 32. For reasons not fully understood, MEH disproportionally affects specific populations of indigenous groups around the world. After the first reports in Native Americans, the epidemiology of the disease has been described in different geographical regions mainly related to particular indigenous populations, the majority of the studies are clinical case reports, but the biological determinants are still unknown. Some suggested risk factors include chronic irritation caused by smoking, a galvanic current, vitamin A deficiency, and/or a familial-genetic predisposition; however, the scientific evidence is not solid due the scarcity of case-control studies or longitudinal cohorts. In light of the evidence, further study of the pathology of MEH should be considered and proper clinical trials for effective treatments should be designed. The disease warrants further study as it is considered as neglected by research and it affects rural/remote population groups usually living in adverse socioeconomic conditions.

## 1 Introduction

MEH or Heck´s disease is an infrequent pathology of the oral mucosa etiologically linked to human papillomavirus low-risk types 13 or 32. The disease is characterized by papulonodular lesions mainly present in lips, inner cheeks, tongue and occasionally the palate; lesions have a smooth surface and are resilient in appearance, and multiple lesions are distributed resembling a cobblestone (García et al., 2011). Patients do not often refer complain of these symptoms, but often complain of repetitive injuries and bleeding due to mastication and aesthetic concerns. The disease affects certain ethnic and indigenous groups with a much higher frequency and Latin America represents one of the geographical regions from which reports of the disease are more common. Several hypotheses have been proposed for the development of the disease, however, the biological determinants are still unknown. This minireview will concisely present the available data on the epidemiology and the hypothesized risk factors associated with this infectious disease and will discuss the knowledge gaps on the mechanisms that lead to the development of the pathology that merit further study.

## 2 Subsections relevant for the subject

### 2.1 Clinical features

MEH is characterized by single or multiple well-delimited sessile lesions which morphologically are round elevations (nodules or papules) with a smooth or irregular not-keratinized surface. The diameter of these lesions varies from 1 to 5 mm and multiple lesions tend to coalesce forming large cobblestones-like areas of the same color of the oral mucosa. A relevant characteristic to differentiate MEH lesions from common warts is that they first disappear when the mucosa is stretched and appear again when the tension is removed (Guevara et al., 2003; De Andrade Santos et al., 2007; Guerra et al., 2007; García et al., 2011; Ledesma-Montes et al., 2012).

**Figure 1:**
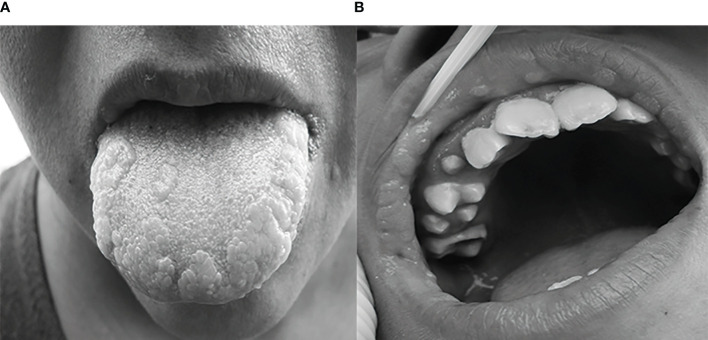
Typical appearance of MEH lesions in a Mayan community, **(A)** lesion in an adult woman’s tongue **(B)** lesion in a boy’s upper lip. Modified from (González-Losa et al., 2011), copyright retained by same authors of this article.

Although the lesions can appear anywhere on the oral mucosa, the most frequently affected anatomical sites are the lower and upper lips, tongue, and hard palate (**Figure 1**). Nodules and papules grow slowly. It is considered a self-limited disease because, in most cases, lesions resolve spontaneously. However, lesions may persist for many years (Guevara et al., 2003; Segura-Saint-Gerons et al., 2005; Navarro, 2006; García et al., 2011).

The histopathological features of MEH lesions are consistent with HPV infections: koilocytes are present (cells with hyperchromatic nuclei with irregular contours and a perinuclear halo), there is visible thickening of the epithelial crests, acanthosis, parakeratosis, and elongation and anastomosis of the rete ridges (Bendtsen et al., 2021). The presence of inflammatory cells in the lesions has been documented (de Castro et al., 2016). Patients generally do not have symptoms, but many complain of repetitive injuries due to mastication with occasional bleeding and aesthetic discomfort.

#### 2.1.1 Diagnosis and treatment

Diagnosis is mainly clinical, identifying the pathognomonic features requires proper training and it is generally not necessary to perform histopathological or virological confirmation.

Treatment options are limited and vary from topical to surgery. In the case of solitary lesions, removal of the tissue by surgery or cryotherapy may be the chosen approach. However this may be too invasive for young patients, not readily available in all settings, or too costly. Laser approaches have also been tested (Nallanchakrava et al., 2018; Sarabadani et al., 2022). In cases with extensive areas of oral mucosa covered by lesions, the surgical approach is not plausible and topical treatments have been proposed. Limited evidence on the benefits of the use of podophyllin, trichloroacetic acid, and imiquimod have been reported (Yasar et al., 2009; Lorduy et al., 2018). Imiquimod 5% cream topical application is an attractive option because of no recurrence of lesions after one year of follow-up and apparent lack of important side effects (Yasar et al., 2009), nevertheless the evidence is still scarce and potential side effects, risks and benefits should be evaluated in clinical trials.

Patients may opt to not undergo treatment (Guledgud et al., 2019). Although it is a benign condition associated with non-oncogenic HPV genotypes (as presented in following section), in case of no regression of the lesions, it is necessary to perform periodic clinical evaluations to detect possible malignant transformations in a timely manner. This is especially relevant among patients who smoke and/or consume alcohol.

### 2.2 Viral agents in the etiology of MEH

The viral etiology of MEH is well recognized, linked to particular human papillomavirus (HPV) types, namely HPV13 and 32 The taxonomy of the Papillomaviridae family includes large genera groups named after the Greek alphabet, and numerous genotypes denoted by Arabic numbers (Bernard et al., 2010). The genotypes belonging to the Alphapapillomavirus are clinically (although not taxonomically) classified in respect to their oncogenic potential as “high risk” and “low risk” types. HPV 32 and 13 are considered as low-risk types because they are not usually detected in cancer tissues and the lesions they cause are generally benign. In spite of belonging to different phylogenetic clades within the Alphapapillomaviruses (see PaVe site by Van Doorslaer et al (Van Doorslaer et al., 2017)), both genotypes show a strong tropism for oral mucosa and are unfrequently found in genitalia (Castro et al., 2011).

MEH was first described more than a century ago and the hypothesis of its viral etiology dates from around 50 years ago. Studies linking the two led to the first isolation of HPV13 viral genome in 1983 (Pfister et al., 1983). Later, HPV32 was identified as an additional etiological agent for MEH (Beaudenon et al., 1987).

However, despite many decades passing since their discovery, reports describing the biology of MEH viral agents are still very limited.

What is known about HPV32 genome is that it is 7.96 kb in length, with 41% of GC content and 6 annotated genes and 6 proteins. The complete genomic sequences are available for HPV32 in the NCBI public repository with three Genbank accessions (NC_001586.1, X74475.1, KT236450.1), with Refseq NC_001586 being the reference sequence (Delius and Hofmann (1994)). No population sets or phylogenetic analyses are reported for HPV32.

A previous, complex analysis of virus-host interactions of E6 and E7 proteins from 11 HPV genotypes included those from HPV32. This study evaluated possible *in vitro* interactions between HPV proteins with around one hundred cellular targets. It is one of the few reports about HPV32 biological features and sheds light on some putative biological determinants of viral pathogenicity and tropism (Neveu et al., 2012). However, *in vitro* observations of protein interactions could not correspond to *in vivo* behavior, so the possible implications must be considered with caution, and merit further study,

In the case of HPV13, the genome is length is 7,880 bp, with 39.4% of GC content, 8 annotated genes and 8 proteins. Three complete genomes are available (Genbank accessions MT068446.1, DQ344807.1, X62843.1), and there is no reference genome, as the previous RefSeq NC_075257.1 has been suppressed at Genbank; nevertheless, X62843 is considered as reference in PaVe database (van Ranst et al., 1992). Only one population set from partial L1 gene sequences has been published worldwide (Cetina-Cetz et al., 2022). To our knowledge, there are no available studies on biological properties of HPV13 viral products.

This scarcity of information is striking in light of the overwhelming amount of data available for other papillomavirus types, such as HPV16, the most popular oncogenic genotype, which comprises more than 11,700 genomic sequences in Genbank, corresponding to more than 4,200 complete genomes and partial-almost complete sequences (https://www.ncbi.nlm.nih.gov/datasets/taxonomy/333760/).

It is important to notice that most of the commercial HPV genotyping kits do not contain probes for HPV13 or HPV32, so these genotypes remain underdiagnosed.

### 2.3 Epidemiology of MEH

MEH occurs mainly in indigenous communities, for reasons still to be determined. MEH is a disease reported in diverse ethnic groups, the majority of which live in the Americas. In 1965 Archard described multiple lesions in the oral mucosa in Native Americans in New Mexico and coined the term focal epithelial hyperplasia, as reported by Gonzalez-Lopez (Gonzalez-López, 2000).

However, in Colombia, “intraoral warts” had been previously described in Caramatas and Katios in 1856 and 1960 respectively (Rosa et al., 2003). Witkop and Niswander (1965), recorded 11 cases: 7 in Xavantes from Brazil, 2 in Ladinos from El Salvador and 2 in Mayas from Guatemala.

Guevara et al. (2003) reported a high prevalence of the pathology (38.1%) in 3877 schoolchildren in rural Peru. In 2006, MEH was reported in Sanema and Yekuana indigenous children from Venezuela (Navarro, 2006) and in Amazonian indigenous groups from Brazil (Borborema-Santos et al., 2006).

A study performed in Colombia, which included 138 schoolchildren, found a 13% prevalence and identified genotypes 55 and 13 in the lesions (González et al., 2005). Later, Cuberos et al (Cuberos et al., 2006), studied the antibodies against HPV13 in the same population, reporting 58% seroprevalence in children with lesions and 33% in children without lesions.

According to González-López (Lopez-Villanueva et al., 2011), the first case in Mexico was recorded in 1971, first in Mexico City and later in Puebla. In Mayan communities in Yucatan, Mexico, our research group has widely documented the presence of MEH associated to HPV13 in children and adults (Lopez-Villanueva et al., 2011). Similarly, in an indigenous community from Chiapas (Mexico) HPV13 was the most prevalent HPV type found in oral cavity with or without lesions (de la Garza-Ramos et al., 2020).

Despite the fact that the majority of cases were initially recognized in indigenous groups from the Americas, several cases have been reported in Africa (Nartey et al., 2003) and countries around the world such as in Greece (Bassioukas et al., 2000), Iran (Mansouri et al., 2015), Saudi Arabia (Al Ameer et al., 2020), amongst others (**Table 1**).

**Table 1:** Distribution of MEH cases from this mini review by geographic location, age and ethnicity.

Location	Ages	Ethnicity (or community group)	Reference
Brazil	11 years	Brazilian, not specified	(Rosa et al., 2003)
	6 to 18 years	Xavantes	(Witkop and Niswander, 1965)
	3 to 17 years	Central Amazonian indigenous	(Borborema-Santos et al., 2006)
Colombia	5 to14 years	Emberá-Chamí community	(González et al., 2005)
	4 to 21 years	Emberá-Chamí community	(Cuberos et al., 2006)
El Salvador	5 to 15 years	Ladinos	(Witkop and Niswander, 1965)
Guatemala	6 to 15 years	Maya	(Witkop and Niswander, 1965)
Peru	5 to 20 years	Mórrope, Lambayaque rural community	(Guevara et al., 2003)
Venezuela	0 to79 years	Sanema and Yekuna	(Navarro, 2006)
Mexico	4 to 73 years	Maya	(Lopez-Villanueva et al., 2011)
	3 to 71 years	Maya	(González-Losa et al., 2011)
	3 to 50 years	Mexican, not specified	(Lama-Gonzalez et al., 2012)
	6 to 18 years	Mazahua and Mestizo	(Gonzalez-López, 2000)
	6 to13 years	Mexican, not specified	(Aguilar- Jiménez et al., 2023)
Ghana	4 to 12 years	Sub-Saharan African	(Nartey et al., 2003)
Greece	<18 years	Caucasian	(Bassioukas et al., 2000)
Iran	35 years	Iranian, not specified	(Mansouri et al., 2015)
Saudi Arabia	21 years	No data	(Al Ameer et al., 2020)

The disease is more frequent in children and adolescents; however, infected individuals without apparent lesions may also play an important role in transmission. In Maya from Yucatan Peninsula (Mexico) we have reported MEH lesions associated with HPV13, as well as subclinical infections with the same genotype within family groups, highlighting the importance of viral carriers of any age within households (González-Losa et al., 2011; Lopez-Villanueva et al., 2011; Lama-Gonzalez et al., 2012). **Supplementary Figure S1**.

It should be noted that sexual transmission is not implicated in this disease and the most likely mode of transmission is close contact between infected peers or family members. Our group has shown evidence to propose saliva as a vehicle of transmission within households (Lopez-Villanueva et al., 2011).

HPV13 is the most frequent genotype in MEH and has been identified as the sole etiological agent amongst the Maya in Mexico. Other genotypes are very rarely associated, a recent report of an outbreak in northern Mexico reported multiple genotypes in 22 cases of the disease in schoolchildren (Aguilar- Jiménez et al., 2023), however, the genotypes present may not necessarily be causal agents of the clinical manifestation. Rare reports include high-risk types and describe the lesions as potentially malignant (Prabhat et al., 2013).

HPV32 has been reported associated to MEH less frequently, although some particular reports have insisted that it may have been overlooked (Khanal et al., 2015).

An important systematic review recently published emphasized numerous reports of the disease in the European region (Sethi et al., 2022), however, the methodology used by the authors dilutes the burden of the disease in Latin America, because the review focused on single-case reports only, published exclusively in English.

### 2.4 Possible risk factors or determinants

Epidemiological determinants may vary geographically. However despite little strong available evidence, some risk factors have been proposed for MEH.

Females have been reported to be more frequently affected by the disease (Agnew et al., 2017), but these conclusions are disputed because of a lack of consistency amongst several reports. Younger age may be considered a risk factor, as the lesions are found mainly between ages 5 and 15. The disease has also been reported in adults, though less frequently (de Castro et al., 2016).

Some reports and case series have linked the disease to social disadvantages which has led to the hypothesis that poverty may be a risk factor. Indeed, a study by our research group found a higher prevalence in children from a low-income elementary school (22%) in comparison to students from middle-income elementary school (2%) (Zavala-Garcia et al., 2017).

In a large analysis performed in aboriginal Australians, it was reported that the only variables associated with increased risk of oral HPV infection were residing in a nonmetropolitan location compared with metropolitan residence (Jamieson et al., 2021) and being a member of an indigenous ethnic group when compared to the general Australian population. In both cases, the researchers found a disproportionately high prevalence of infections with the HPV13 and HPV32 genotypes (Antonsson et al., 2014). The authors discuss several indicators of social disadvantage in the affected populations (Jamieson et al., 2021). However, there are no available studies analyzing these particular social problems with an objective instrument.

Because of the geographical and ethnical grouping of MEH cases, a genetic predisposition has been suggested, however very little biological evidence is available. The sole reported genetic association with the presence of the disease is allele HLA-DR4 (DRB1*0404) in a Mexican population (García-Corona et al., 2004), an allele frequently found in Native American descendants.

Future studies must focus on the characterization of the genetic determinants involved, including ethnicity, ancestry, additional HLA alleles, cytokine and other immunological determinants related to the anti-HPV cellular response.

## 3 Discussion

MEH is still considered a rare disease, with limited information available worldwide, despite the evidence of its presence in many countries. The majority of reports are description of clinical cases, and only a hand-full of research groups, mainly from Latin America, have performed an active search for affected populations to investigate and characterize the disease’s determinants and epidemiology.

Although MEH is considered a reversible disease, its natural history is largely unknown, and longitudinal studies are lacking. Given the evidence of infection carriers longitudinal cohorts are needed to discover the factors associated with persistence and elimination, as well as the evolution of the lesions. One of the largest cohorts studying HPV13 and 32 infections included 558 indigenous Australians of 20.4% incidence and 13.3% persistence at 12-month follow-up; unfortunately, clinical data was not included and lesions were not recorded by the authors (Jamieson et al., 2021).

MEH transmission dynamics are still unknown. The presence of the virus in saliva is a fact and could represent the main transmission route including contaminated shared objects (Lopez-Villanueva et al., 2011). Prevention and control strategies can only be established after clearly determining the transmission dynamic within families and communities.

To date, MEH is considered a multifactorial pathology, however, there are still no solid data to support this theory. The only case-control study by Cuberos et al. (2006) demonstrated HPV13 is strongly associated with the disease.

In the case of cervical cancer, HPVs have been widely documented as causal agents after many decades and thousands of studies. This library of knowledge has helped define the etiology of cervical cancers in the clinic. Indeed, the so called risk factors for cervical cancer are in fact the risk factors for HPV infection. In the case of MEH, despite insufficient evidence to draw conclusions, we consider a similar theory that the disease is caused by a viral infection as unique cause. We find it likely that the other hypothesized risk factors (living in marginalized conditions, genetic predisposition, nutritional deficiencies, etc.) are, in fact, determinants that make the individuals vulnerable to acquire the infection. With the limited evidence to date, more research should be encouraged to understand the possible risk factors or associated cofactors, but it is clear that the viral agents are the etiological cause of MEH.

Finally, it should be highlighted that, although not listed as a neglected disease, MEH is indeed neglected by research and primarily affects neglected populations. The knowledge gaps urge research attention to better understanding on the natural history of this understudied disease and for prevention and treatment options for the affected populations.

## Author contributions

LC: Conceptualization, Writing – original draft, Writing – review & editing. MG: Conceptualization, Writing – original draft, Writing – review & editing.
