# Supplementary figures and images for: Multifocal epithelial hyperplasia: an understudied infectious disease affecting ethnic groups. A mini review
Source: Front Cell Infect Microbiol. 2024 Jul 25;14:1420298. doi: 10.3389/fcimb.2024.1420298 (PMC11306162; doi:10.3389/fcimb.2024.1420298)

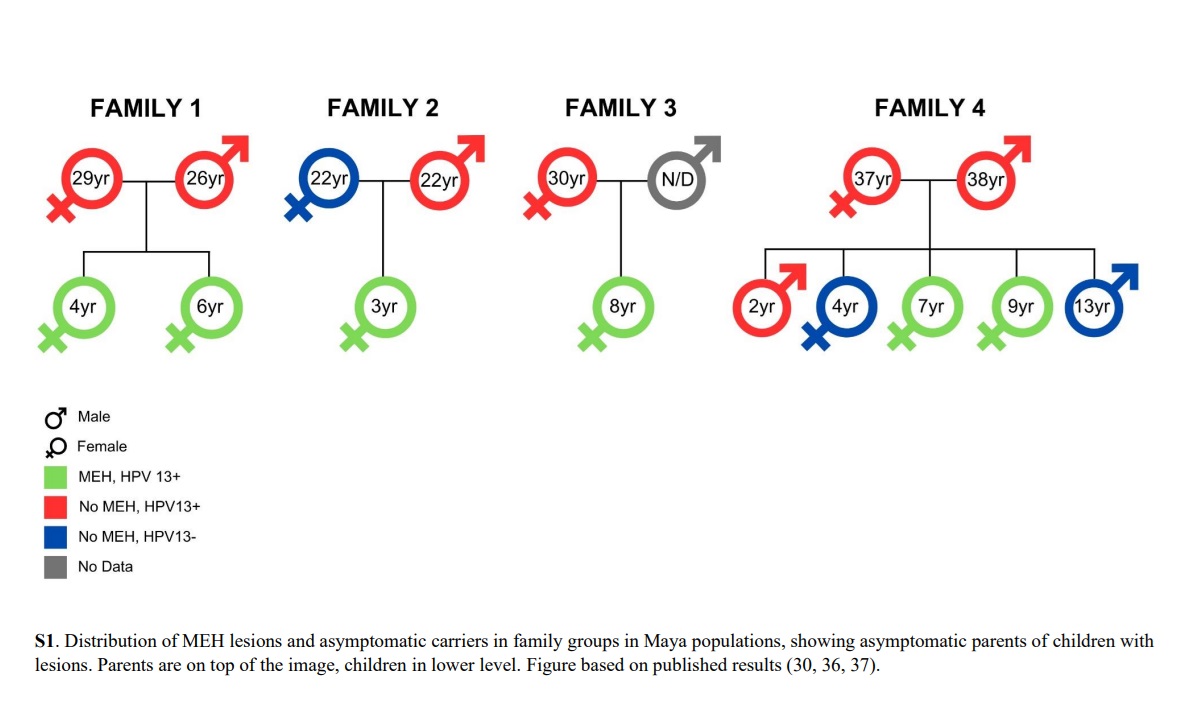

Supplement: Supplementary file 1 [file Image_1.jpg]
